# Strain and Spin-Orbit Coupling Engineering in Twisted WS_2_/Graphene Heterobilayer

**DOI:** 10.3390/nano11112921

**Published:** 2021-10-31

**Authors:** Cyrine Ernandes, Lama Khalil, Hugo Henck, Meng-Qiang Zhao, Julien Chaste, Fabrice Oehler, Alan T. Charlie Johnson, Maria C. Asensio, Debora Pierucci, Marco Pala, José Avila, Abdelkarim Ouerghi

**Affiliations:** 1 Centre de Nanosciences et de Nanotechnologies, CNRS, Université Paris-Saclay, 91120 Palaiseau, France; cyrine.ernandes@universite-paris-saclay.fr (C.E.); lama.khalil@universite-paris-saclay.fr (L.K.); hugo.henck@gmail.com (H.H.); julien.chaste@c2n.upsaclay.fr (J.C.); fabrice.oehler@c2n.upsaclay.fr (F.O.); debora.pierucci@c2n.upsaclay.fr (D.P.); marco.pala@c2n.upsaclay.fr (M.P.); 2 Department of Chemical and Materials Engineering, New Jersey Institute of Technology, 138 Warren Street, Newark, NJ 07103, USA; mz24@njit.edu; 3 Department of Physics and Astronomy, University of Pennsylvania, 209S 33rd Street, Philadelphia, PA 19104, USA; cjohnson@physics.upenn.edu; 4 Materials Science Institute of Madrid (ICMM), Spanish Scientific Research Council (CSIC), Cantoblanco, 28049 Madrid, Spain; mc.asensio@csic.es; 5 MATINEE, CSIC Research Associated Unit between the Institute of Materials Science of the Valencia University (ICMUV) and the ICMM, Cantoblanco, 28049 Madrid, Spain; 6 Synchrotron-SOLEIL, Université Paris-Saclay, Saint-Aubin, BP48, 91192 Gif sur Yvette, France

**Keywords:** twisted heterobilayer, van der Waals materials, spin-orbit coupling, band structure

## Abstract

The strain in hybrid van der Waals heterostructures, made of two distinct two-dimensional van der Waals materials, offers an interesting handle on their corresponding electronic band structure. Such strain can be engineered by changing the relative crystallographic orientation between the constitutive monolayers, notably, the angular misorientation, also known as the “twist angle”. By combining angle-resolved photoemission spectroscopy with density functional theory calculations, we investigate here the band structure of the WS_2_/graphene heterobilayer for various twist angles. Despite the relatively weak coupling between WS_2_ and graphene, we demonstrate that the resulting strain quantitatively affects many electronic features of the WS_2_ monolayers, including the spin-orbit coupling strength. In particular, we show that the WS_2_ spin-orbit splitting of the valence band maximum at K can be tuned from 430 to 460 meV. Our findings open perspectives in controlling the band dispersion of van der Waals materials.

## 1. Introduction

The successful fabrication of monolayer graphene (Gr) by mechanical exfoliation has triggered an intense interest in transition metal dichalcogenides (TMDs, e.g., MX_2_ with M = W, or Mo and X = S, Se, or Te), which are two-dimensional (2D) semiconductors whose optical and electronic properties can be tailored as a function of the number of layers and the stacking orientation [1]. More interestingly, assembling TMD monolayers into van der Waals (vdW) heterostructures can further modulate these properties through the choice of materials and the relative alignment between the monolayers [2]. While covalent heterostructures are governed by a fixed lattice mismatch and interlayer misorientation, vdW heterostructures present an additional degree of freedom in the arbitrary rotation angle between the layers, referred to as the “twist” angle. For small twist angles, a long wavelength periodic strain modulation arises, known as the Moiré superlattice [3]. The formation of Moiré superlattices between 2D materials directly alters the electronic band structure [4], and sometimes creates spectacular arrangements [5]. Notably, specific “magic” twist angles have been associated to unique dispersion relations with localized flat bands in bilayer graphene and TMDs [6]. An experimental study using scanning tunneling microscopy has recently evidenced these quantum-confined electronic states near the band edges for aligned MoS_2_/WSe_2_ heterobilayers [7]. Experimentally, the range of “magic” angles is much wider in TMD/TMD heterostructures than in twisted bilayer graphene (Gr/Gr), for which the flat band configuration only exists within ±0.2° of the magic angle [3,5]. New variants of twisted hybrid heterostructures have also been proposed, in which the monolayers are made of distinct 2D materials (e.g., TMD/Gr) instead of sibling TMD crystals. In such TMD/Gr heterostructures, several electronic features can be improved with respect to the corresponding TMD monolayer structure, as in the case of WS_2_/Gr heterobilayers, which exhibit remarkable advantages compared to monolayer WS_2_ [8,9,10]. While MX_2_ monolayers are generally limited by a relatively low carrier mobility and photosensitivity, the MX_2_/Gr heterobilayers often offer much improved values, which leads to direct applications in photodetector and transistor devices [11]. Finally, recent theoretical and experimental studies have reported that strong spin-orbit interactions (SOI) can be induced in graphene using MX_2_/Gr heterobilayers [12,13,14]. The SOC prevents novel quantum states from emerging, such as the quantum anomalous Hall state. On the other hand, owing to their strong SOC, MX_2_ provide an ideal platform to increase the SOC in graphene by the proximity effect [14,15,16].

However, less attention has been paid to the modification of the TMD band structure in these heterobilayers [15]. In particular, the impact of the “twist” angle variations on the band structure of WS_2_/Gr heterobilayers has never been investigated experimentally. Nanoscopic angle-resolved photoelectron spectroscopy (nano-ARPES) is a powerful technique used to experimentally study and visualize the band structure around the valence band edge of twisted heterobilayers. Here, using nano-ARPES, we investigate the electronic structure of WS_2_/Gr at different twist angles. For all our measured twist angles, we observe that the graphene bands are n-doped, and that the graphene-derived Dirac point is located within the WS_2_ bandgap. We experimentally show that the strength of spin-orbit coupling (SOC) in the WS_2_-related valence band depends on the interlayer twist angle between WS_2_ and graphene, and that the SOC value is 30 meV higher than the reference monolayer value.

## 2. Materials and Methods

The WS_2_/SiO_2_ samples were grown by chemical vapor deposition (CVD) in a 1” quartz tube furnace. The growth substrate (SiO_2_/Si(001) was placed in the center of the furnace and heated to 800 °C. A 25 mg sulphur pellet was placed on a piece of silicon and positioned upstream in the furnace such that its temperature was approximately 150 °C. Carrier gas (500 sccm N_2_ and 15 sccm H_2_) was used to bring sulphur vapor into the furnace for a 30 min growth period. A PMMA-assisted method was employed to transfer the CVD-grown WS_2_ onto epitaxial monolayer graphene on SiC(0001) [17]. Typical single-crystal domains with an equilateral triangle shape were obtained by the CVD growth procedure. The WS_2_ domains transferred onto the graphene retained their triangular shapes, with lateral sizes of ~20 to ~200 µm. To further clean the surface and interface of the WS_2_/Gr, we annealed the samples at 350 °C for 120 min in UHV (base pressure below P~10^−10^ mbar).

The µ-PL/Raman measurements were conducted at room temperature, using a commercial confocal Horiba micro-Raman microscope (HORIBA, Palaiseau, France) with a 100× objective, and a 532 nm laser excitation [18]. The nano-ARPES measurements (spot size about 600 nm) were conducted at the Antares beamline of the SOLEIL synchrotron (Saint-Aubin, France). We used linearly polarized photons of 100 eV and a hemispherical electron analyzer scienta R4000 with horizontal slits to allow band mapping. All nano-ARPES experiments were performed at a low temperature of 70 K, and the energy resolution was about 10 meV.

The band structure calculations were realized with the QUANTUM EPSRESSO code [19]. We adopted fully relativistic pseudopotentials and performed noncollinear simulations to include the spin-orbit interaction. For the exchange-correlation term, we considered both the PBE and the HSE hybrid functionals [20] to better estimate the band-gap energy. The self-consistent solution was obtained by adopting a 10 × 15 × 1 Monkhorst-Pack grid and a cutoff energy of 50 Ry. A vacuum space of 20 Å along the vertical direction was used to minimize the interaction between two adjacent sheets. Cell parameters and atomic positions were relaxed according to a convergence threshold for forces and energy of 10^−3^ and 10^−4^ (a.u.), respectively.

## 3. Results and Discussion

In the absence of other direct experimental investigations on the band structure of twisted WS_2_ monolayers on graphene, the present nano-ARPES study reveals original strain-related changes in the band dispersion and SOC strength of WS_2_/Gr heterostructure. In a previous report, we have proved that it is possible to resolve the bands of WS_2_ flakes transferred on an epitaxial graphene underlayer [17]. In the present work, we followed the same procedure to obtain the WS_2_/Gr heterobilayer. Here, monocrystalline mono- and few-layer WS_2_ flakes were first grown on a SiO_2_/Si substrate using a CVD technique (see Materials and Methods), and then transferred onto a Gr/SiC substrate [21]. To ensure a proper cleaning of interfaces, the samples were annealed at 350 °C in vacuum before the nano-ARPES measurements.

**Figure 1:**
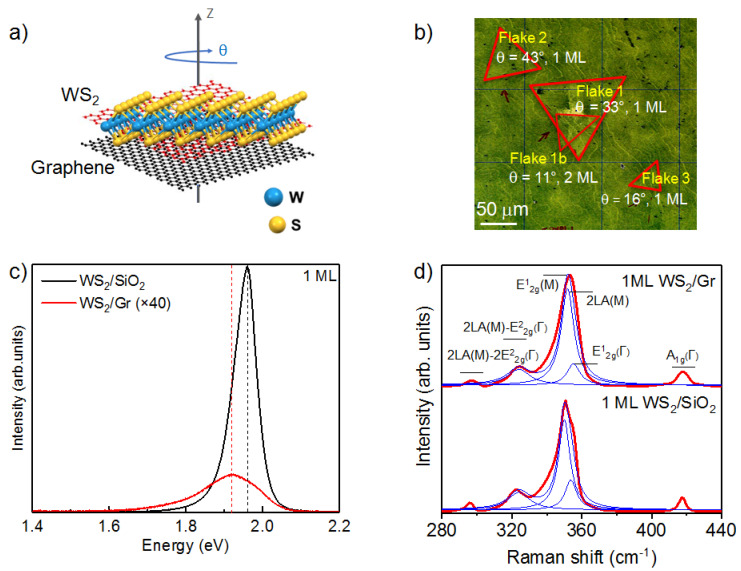
Structural and electronic properties of a WS_2_/Gr heterostructure: (**a**) 3D view of graphene on top of monolayer TMDs. Here, θ is the twist angle between graphene and the TMD layers; (**b**) Optical image of the WS_2_ transferred onto the graphene layer; (**c**,**d**) Room temperature PL and micro-Raman spectra of Flake 1 before (1 ML WS_2_/SiO_2_), and after (1 ML WS_2_/Gr), transfer.

A schematic of the atomic structure of the WS_2_/Gr hybrid system, which can present an arbitrary in-plane twist angle, *θ*, between the monolayers, is shown in Figure 1a. Figure 1b presents an optical image of the sample, showing an area with several WS_2_-flake-transferred graphene. The overall coverage of the graphene surface by WS_2_ was approximatively 30%. Because of the poor optical contrast on the SiC substrate, red contours have been drawn to outline the shape of the WS_2_ flakes. As is visible in Figure 1b, the optical image consists of regular triangular WS_2_ flakes, arbitrarily rotated with respect to the underlying graphene substrate. While it is possible to directly estimate the value of the twist angle, *θ*, between the WS_2_ flakes and graphene from the optical image, more accurate values can be obtained using nano-ARPES. To this end, nano-ARPES constant energy maps were recorded outside and inside the WS_2_ flakes. The twist angle, *θ,* was then defined in the reciprocal space as the angle between the $\Gamma K_{Gr}$ and $\Gamma K_{\mathrm{WS}2}$ directions. We obtained values of θ = 33°, 11°, 43°, and 16° for Flake 1, Flake 1b, Flake 2, and Flake 3, respectively.

To investigate the optical properties of the WS_2_ flakes, µ-PL/Raman spectroscopy was carried out at room temperature [22]. The vibration modes of Flake 1 can be obtained by using micro-Raman (μ-Raman) spectroscopy. Figure 1d shows the μ-Raman scattering spectra obtained before and after transferring the WS_2_ flakes onto graphene. For both spectra, we observed the in-plane phonon mode, $E_{2g}^{1}$, and the out-of-plane phonon mode, A_1g_. In particular, the $E_{2g}^{1}$(Γ) mode, observed at 353.8 cm^−1^ for WS_2_/SiO_2_, was redshifted of 2 cm^−1^ with respect to that of WS_2_/Gr. Similarly, the A_1g_(Γ) peak, observed at 417.7 cm^−1^ [23,24] for WS_2_/SiO_2_, also shows a small redshift of about 1 cm^−1^ with respect to that of WS_2_/Gr. These shifts for both modes confirm the interlayer e coupling between WS_2_ and graphene, as seen by the PL measurements. These shifts could also be attributed to a stress reduction when WS_2_ is transferred onto the graphene substrate [17]. Beside the A_1g_ and $E_{2g}^{1}$ modes, both Raman spectra present a series of overtone and combination peaks. For WS_2_/SiO_2_, the second order Raman peak, 2 LA(M), relative to the longitudinal acoustic phonons at the M-point in the Brillouin zone (BZ), and the $E_{2g}^{1}\left(M\right)$ mode, are observed at 349.8 cm^−1^ and 350.5 cm^−1^, respectively [25]. These peaks were redshifted of 1 cm^−1^ or 2 cm^−1^, compared to those of WS_2_/Gr, attributable to a strain between the WS_2_ and the graphene substrate. Additional Raman peaks around 296 cm^−1^ and 323 cm^−1^ were combination modes, attributed to the $2LA\left(M\right)-2E_{2g}^{2}\left(\Gamma \right)$ and the $2LA\left(M\right)-E_{2g}^{2}\left(\Gamma \right)$ modes, respectively [8].

Before transferring Flake 1 (1 ML) and Flake 1b (2 ML) onto graphene, we measured their PL spectra on the SiO_2_ substrate (see Supplementary Information, Figure S1). As shown in Figure S1, the PL intensity of Flake 1 is one order of magnitude higher than that of Flake 1b. This is consistent with the literature and indicates that a direct to an indirect bandgap transition occurs when increasing the number of layers of WS_2_ [26]. To study the electronic coupling between 1 ML of WS_2_ and the graphene, we compare, in Figure 1c, 1 ML of the WS_2_ PL spectra acquired on the SiO_2_ and graphene substrate, respectively. We observed a drastic quench of the PL intensity for WS_2_ on graphene, attributed to an interfacial electron transfer from the n-doped graphene substrate to WS_2_ [27,28,29,30,31]. This electron transfer, thus, reflects the electronic coupling between WS_2_ and graphene. From the PL spectrum of 1 ML of WS_2_ on graphene, we also extracted the corresponding optical bandgap value of 1.95 eV, in agreement with previous experimental reports [32,33,34,35,36]. We noticed, as well, a bandgap decrease of about 40 meV in the case of the WS_2_/Gr. The decrease of the PL intensity and the bandgap can be explained by a charge transfer and strain induced by the graphene layer. The possible band structure and bandgap change in single layer WS_2_ could be translated by the orientation angle of the flakes with respect to the graphene substrate and may induce a variation of the properties of WS_2_. Thus, despite the clean interface between WS_2_ and graphene [8], both the PL and µ-Raman results prove that the two layers are coupled electronically.

**Figure 2:**
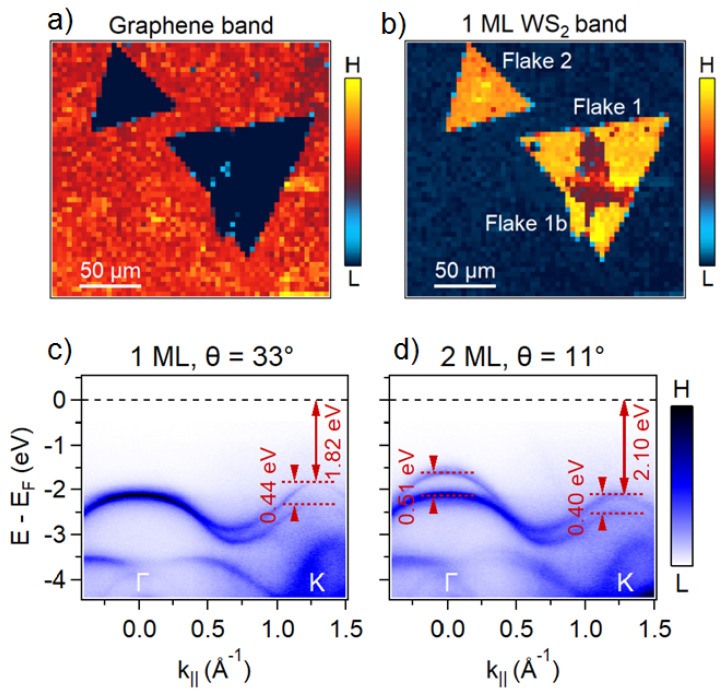
Comparison of the electronic band structure between single and bilayer WSe_2_: (**a**,**b**) Spatially resolved ARPES map of the π graphene band and the 1 Ml WS_2_ band; (**c**,**d**) ARPES map of single and bilayer WS_2_ along the ΓK high-symmetry directions.

We now turn to the nano-ARPES characterization of the WS_2_/Gr heterostructures to study the coupling between the WS_2_ and the graphene substrate. We obtained a sharp ARPES signal on the graphene and on each individual flake, which we associated with the good material quality and sample fabrication. Combining the total valence band mapping in the reciprocal space, and the spatial information in the direct space, we were able to extract and to localize, with nanometric spatial resolution, the different contributions of each material in the final electronic band structure. In Figure 2, we can thus identify and separate the following dispersion relations: the graphene π bands, and the monolayer (1 ML) WS_2_ and the bilayer (2 ML) WS_2_ valence bands (Figure 2a,b). By integrating the photoemission intensity in an energy window centered around the valence band maximum (VBM), while scanning the sample in real space along the two in-plane directions, we obtained spatial maps and morphological information. Figure 2a is centered on the graphene π band’s energy, so that high-intensity signal (red color) shows the extent of the continuous graphene underlayer, while the region covered by WS_2_ crystals appears as low signal (blue color). Conversely, the map in Figure 2b integrates around the WS_2_ VBM energy and shows three distinct regions: high (red), medium (yellow), and low (blue) intensities, which correspond to 2 ML WS_2_, 1 ML WS_2_, and graphene, respectively. These two WS_2_ and graphene nano-ARPES spatial maps (Figure 2a,b) further confirmed the staking sequence of our heterostructure, as deduced from the previous optical characterizations (PL, Raman) in Figure 1. Each of these regions (Gr, 1 ML WS_2_, and 2 ML WS_2_) displayed a uniform intensity, attributed to the homogeneity of the electronic properties. The single and robust Dirac cone at the K high-symmetry points (at k_||_ = 1.703 Å^−1^) confirms that the graphene inside the heterostructure preserves its Dirac linear dispersion, and the massless relativistic character of the graphene monolayer carriers close to the Fermi level. The analysis of the π band dispersion, inside and outside, determines a Fermi velocity $v_{F}\sim \;1.1\times 10^{6}$ m/s. The neutrality level (the Dirac point) in the band structure of the graphene was observed at a binding energy of about 0.3 eV below Fermi level. The electron doping level can be estimated to about 9 × 10^12^ cm^−2^ (see Supplementary Information, Figure S2) and is rather high in contrast to isolated monolayer graphene. This doping is explained conventionally by donor-like states associated with the interface layer between the graphene and the SiC(0001) substrate that overcompensate for the polarization doping from the SiC substrate [30,31,32,33,34].

We then extracted and analyzed the electronic band parameters along the reciprocal Γ-K direction of the WS_2_/Gr heterostructure (Flake 1 and Flake 1b), depending on the thickness of the WS_2_ flakes. The number of branches at the Γ point agreed with the number of layers: one for the monolayer, and two for the bilayer. From the ARPES data in Figure 2c,d, we clearly detect the layer-dependent band structure evolution. In particular, the change from the 1 ML WS_2_/Gr to the 2 ML WS_2_/Gr moves the VBM from the K point for the 1 ML WS_2_/Gr (1.82 eV binding energy), to the Γ point for the 2 ML WS_2_/Gr (binding energy of 1.62 eV). We thus expect that the 1 ML of WS_2_/Gr will show a direct bandgap, VBM, and conduction band minimum (CBM) at the K point, while the 2 ML WS_2_/Gr is indirect, with the VBM located at Γ, and the CBM at K. Therefore, the overall variation of the WS_2_/Gr band structure with the WS_2_ thickness (1 ML, 2 ML) is in agreement with DFT theory [5]. Up to now, our results corroborate closely to those of Coletti et al. on the photoemission of an epitaxial-aligned (θ = 0°) 1 ML WS_2_/Gr/SiC heterostructure [9], for which the relatively weak WS_2_/Gr interaction causes the constitutive monolayer of the heterostructure to retain most of the features of their initial band structure. However, a more detailed look at the TMD-related bands near K reveals important details. In WS_2_, as in other TMD materials, the strong spin-orbit coupling (SOC) of transition-metal *d* orbitals leads to an energy splitting (Δ_SOC_) of the valence bands at the six corners (K, K’ points) of the BZ. This large Δ_SOC_ value mainly originates from the hybridization between the W *d_xy_* and *d_x2-y2_* and the S *p_x_* and *p_y_* bonding states [35]. Using the experimental nano-ARPES dispersion at K, we can, thus, directly measure the value of Δ_SOC_ in our samples. For monolayer WS_2_, this value is predicted to be 430 meV [5], but Coletti et al. [9] already report a much higher value of Δ_SOC_ = 462 meV for epitaxial (θ = 0°) WS_2_/Gr/SiC [9], which suggests that there is some degree of interaction between the WS_2_ and the Gr underlayers. The band splitting in our sample is about 1 ML WS_2_/Gr (Flake 1, Δ_SOC_∼440 meV), and 2 ML WS_2_/Gr (Flake 1b, Δ_SOC_∼400 meV) heterostructures. As those two structures differ in ML thickness (1 vs. 2 ML), and twist angle θ (33° vs. 11°), the Δ_SOC_ difference cannot be unambiguously attributed to the twist angle effect.

**Figure 3:**
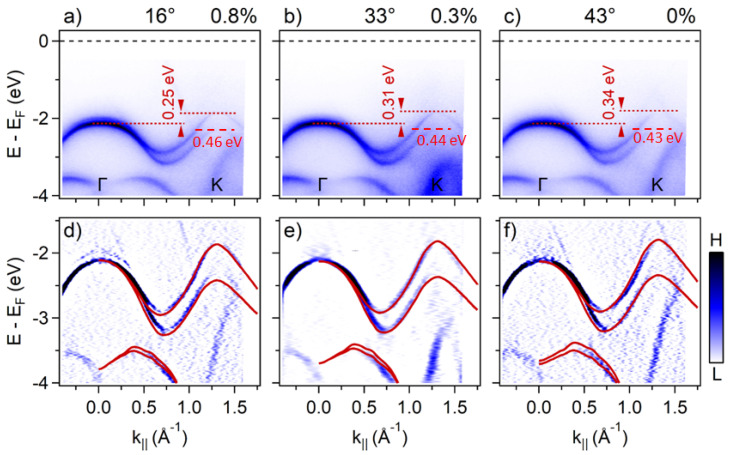
Evolution of the electronic band structure of the WS_2_/Gr heterobilayer at various twist angles: (**a**–**c**) ARPES band map along GK of the WS_2_ layer, with twist angles of 16, 33, and 43°, respectively; (**d**–**f**) Corresponding second derivative spectra of the (**a**–**c**) panels on which the calculated band structure obtained by HSE06 (red lines) is superimposed. The tensile biaxial strain used in the simulations is marked next to the experimental twist angle.

To isolate the impact of the twist angle, we now focus on 1 ML WS_2_/Gr heterobilayers at K. Compared to epitaxial vdW heterostructures, which are limited to θ = 0° but always present a coupling between the layers, our transferred WS_2_/Gr heterobilayer can sample arbitrary twist angles, but the interlayer coupling needs to be confirmed. Figure 3a–c present the experimental valence band structures acquired by nano-ARPES for θ = 33° (Flake 1), 43° (Flake 2), and 16° (Flake 3), using the naming scheme of Figure 1. We found that the Δ_SOC_ varied widely, starting from 460 meV at θ = 16°, down to 440 meV near θ = 33°, and about 430 meV for θ = 43°. Additionally, we observed that the energy difference between the VBM at Γ and K, (Δ_ΓΚ_) also varied with the twisted angle. In Figure 3d–f, the second derivative spectra of the experimental data were compared with the DFT calculations obtained by HSE06 (red lines). The main ARPES features are well-reproduced by the calculations for all twist angles, but tensile bi-axial strain must be introduced to obtain the correct Δ_ΓΚ_ spacing. The total variation from the reference theoretical value of 430 meV is significant and confirms that our transferred 1 ML WS_2_ flakes effectively couple to the Gr underlayer. From these measurements, we conclude that the twist angle modulates the SOC strength in the TMD-related band of 1 ML WS_2_/Gr heterobilayers, with positive variations up to 30 meV from the reference theoretical value.

We have considered two hypotheses to explain the observed variation of the SOC in our twisted 1 ML WS_2_/Gr heterobilayers: (i) electronic coupling via charge transfer; and (ii) biaxial strain at the WS_2_/Gr interface [36].

(i) First, we can consider the charge transfer probabilities occurring at specific twist angles as the main contribution to the Δ_SOC_ variations. In the present case, the heterostructure is formed by n-type graphene and n-type WS_2_, which creates a Schottky barrier. The Schottky barrier height (SBH) thus corresponds to the energy difference between the CBM of the WS_2_ band, and the position of the Fermi level in the graphene Dirac cone [4]. Since the graphene layer is n-doped, a charge transfer could occur from the K valley of the graphene to the CBM of the WS_2_. Following [15,16], the interlayer interaction between WS_2_ and Gr is proportional to the transfer integral between the two lattices, which decreases exponentially with the interlayer spacing in real space, but also requires momentum matching in the reciprocal space. As illustrated in Supplementary Information (Figure S3), the positions of three specific k-vectors in the BZ of WS_2_ vary with the twist angle (red circles) and can approach the CBM of WS_2_ (green symbols). It turns out that the interlayer coupling and, hence, the spin-splitting of WS_2_, is maximal when the wavevectors, $k_{1}^{\theta }$, $k_{2}^{\theta }$, and $k_{3}^{\theta }$, approach the CBM of the WS_2_ located in the Q point (middle point between Γ and K) for θ~20°. While this effect may occur in our structures, the expected order of magnitude (a few meV) and angular positions do not match our experiments. Therefore, charge transfer is not the main contributor to the observed Δ_SOC_ variations;
(ii) Mechanical strain is an expected feature of vdW heterostructures, which assemble materials of different lattice parameters. Although the vdW interaction is weaker than covalent bounds, it can still create Moiré superlattices that can affect the material strain and its electronic properties. The superstructure is governed by the lattice mismatch and by the rotational and translational misalignments of the two lattices. Here, the observed angular misalignment, θ WS_2_/Gr, combined with the theoretical lattice mismatch, (*a*_Gr_~2.46 Å, *a*_WS2_~3.16 Å), can create a long-range strain modulation, which can be grossly approximated to a uniform tensile or compressive in-plane strain [37].

Hence, the latter explanation, (ii), seems to better suit our experimental observations. To support this argument, we now compare our experimental ARPES data with band structure calculations computed using DFT. For monolayer WS_2_, a tensile in-plane biaxial strain will decrease the vertical distance between the upper and lower sublayers of the S atoms, which, in turn, reduces the bandgap and affects the Δ_SOC_ at K [36,38]. In the DFT calculations, the biaxial strain is applied by modifying the lattice constant of WS_2_, followed by a structural relaxation to obtain the most favorable atomic positions.

**Figure 4:**
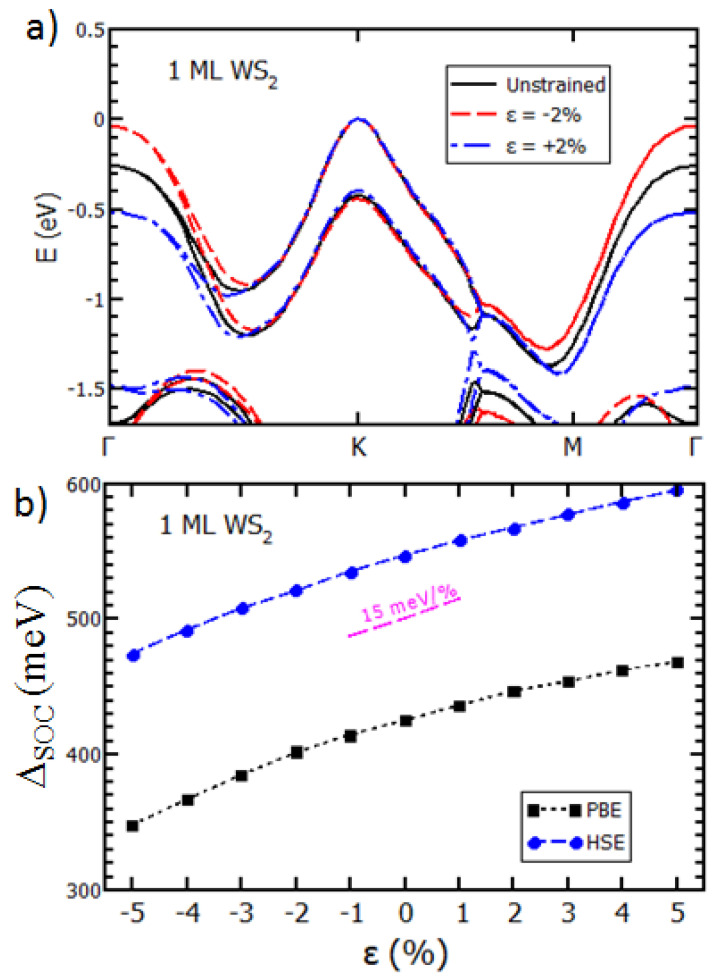
Structural and electronic properties of WS_2_ under strain: (**a**) Band structures of WS_2_ for different strains; (**b**) DFT calculations of the evolution of SOC under strain (PBE and HSE06, respectively).

To further test our calculations, we have performed additional DFT simulations using another exchange-correlation functional (PBE vs. HSE). In addition to the atomic structure, Figure 4a shows the calculated electronic band structure for WS_2_ for the tensile and compressive biaxial strain values (±2%). Although the overall dispersions look similar, there are differences between all the structures. To compare with the experimental ARPES result, we first focus on the top of the valence band near the K point. In agreement with the experiment, our DFT unstrained WS_2_ monolayer is a semiconductor with a 1.58 eV direct bandgap (i.e., VBM and CBM located at K). The Δ_SOC_ value depends on the chosen exchange-correlation functionals (430 meV PBE, 551 meV HSE). We confirm that the Δ_SOC_ of the VBM at K increases with the tensile biaxial strain, but that the rather extreme value of 1% merely increases the Δ_SOC_ by only 15 meV. The opposite variation is observed for compressive biaxial strain (see Supplementary Information, Figure S4). The final inferred Δ_SOC_ variation, +15 meV per 1% in plane tensile strain, is independent of the chosen exchange-correlation functionals (PBE or HSE) and matches the existing theoretical results on WS_2_ and WSe_2_ [36,38]. However, the variation of Δ_SOC_ at K is not the only marker of biaxial strain in the WS_2_ band structure. As shown in Figure 4a, the energy position of the valence band near Γ also varies critically with the in-plane strain, while that near K remains mostly static. Therefore, we can estimate the biaxial strain in the WS_2_ layer by fitting the valence band’s energy position along the Γ−Κ direction. In a first approximation, we simply used the energy difference, Δ_ΓΚ_, and we computed tensile biaxial strains of +0.8% (θ = 16°), +0.3% (θ = 33°), and 0% (θ = 43°). Such strain could arise from the fabrication process during the cooling down of the sample after the 350 °C annealing. From these results, we can recalculate, with DFT (PBE), the strain-corrected Δ_SOC_theo_ values of 440 meV (θ = 16°), 434 meV (θ = 33°), and 430 meV (θ = 43°). Table 1 summarize these results, along with the corresponding experimental Δ_SOC_ data.

Compared with the experimental Δ_SOC_ values, we see that this simple biaxial strain model reproduces the sign and the order of magnitude of the Δ_SOC_ variations. For such large strain values, it is important to check the behavior of the conduction band. Here, the DFT results show the existence of the local minimum of the conduction band along the Γ–K direction (Q point), which should lead from a direct to an indirect bandgap at about 1% compressive strain (see Supplementary Information, Figure S4). Such a drastic change should be clearly visible in the optical characterization (PL), thus implying that only tensile strain was present in our samples. When comparing the charge transfer to the biaxial strain, we conclude that biaxial strain better reproduces the observed change in SOC in the TMD-related band of our WS_2_/Gr heterostructure.

## 4. Conclusions

In summary, we studied the electronic structure, using nano-ARPES, of a 1 ML WS_2_/Gr heterobilayer with different twist angles. Because of the relatively weak coupling between Gr and WS_2_, the valence bands of the heterostructure retained some resemblance to their elementary constituent, but the detailed analysis of the TMD-related bands reveals a large twist-angle dependence. Specifically, the spin-orbit splitting of the valence band maximum at K (Δ_SOC_) can be tuned from 430 to 460 meV. While theoretical modeling combining the biaxial strain in the DFT calculation can reproduce some of the features of the band structure, such extreme Δ_SOC_ variations currently elude our modeling attempts. On the practical side, this work implements yet another possible route for tailoring the band structure of TMD materials: by controlling the twist angle between the TMD/Gr heterojunctions.

## Figures and Tables

**Table 1:** Summary of 1 ML WS_2_/Gr flake properties: experimental twist angle (from ARPES); energy difference of the VBM Δ_ΓΚ_; corresponding computed biaxial strain (as determined from DFT); corresponding theoretical Δ_SOC_theo_ from PBE functional (reference value 430 meV at 0% strain); and experimental Δ_SOC_ measurements.

	Flake #3	Flake #1	Flake #2
WS_2_/Gr twist angle (°)	16°	33°	43°
Valence Δ_ΓΚ_ difference (eV)	0.25	0.31	0.34
Computed biaxial strain (%)	+0.8	+0.3	0.0
Theoretical Δ_SOC_theo_ (meV) at K	440	434	430
Experimental Δ_SOC_ (meV) at K	460 ± 5	440 ± 5	430 ± 5

## Data Availability

The datasets generated during and/or analyzed during the current study are available from the corresponding author on reasonable request.

## Supplementary Materials

The following are available online at https://www.mdpi.com/article/10.3390/nano11112921/s1, Figure S1: Typical Band structure of graphene layer on SiC (0001) (inside and outside of the WS2), Figure S2: (a) Band-structure calculations of the monolayer WS_2_ along the high symmetry points of the Brillouin zone for different stress levels. (b) The evolution of the three wavevectors $k_{1}^{\theta }$**,** $k_{2}^{\theta }$ and $k_{3}^{\theta }$ as a function of the twist angle θ. The hexagons represent the first Brillouin zone of (black) graphene, (red) WS_2_ with θ=0 and (magenta) WS_2_ with θ=20°. The green symbols represent the evolution of the Q points. (c) Distance between $k_{1}^{\theta }$ and Q as a function of θ, Figure S3: Evolution of the band-structure calculations of the monolayer WS_2_ under strain, Figure S4: Evolution of the band-structure calculations of the monolayer WS_2_ under strain.
