# Supplementary material for: Strain and Spin-Orbit Coupling Engineering in Twisted WS2/Graphene Heterobilayer
Source: Nanomaterials (Basel). 2021 Oct 31;11(11):2921. doi: 10.3390/nano11112921 (PMC8625993; doi:10.3390/nano11112921)
Supplement: Supplementary file 1 [file nanomaterials-11-02921-s001.zip › nanomaterials-1448413-supplementary.pdf]

nanomaterials

MDPI

Supplementary Information:

Strain and Spin-Orbit Coupling Engineering in Twisted WS_2/Graphene Heterobilayer

Cyrine Ernandes [1], Lama Khalil [1], Hugo Henck [1], Meng-Qiang Zhao [2], Julien Chaste [1], Fabrice Oehler [1], Alan T. Charlie Johnson [3], Maria C. Asensio [4,5], Debora Pierucci [1], Marco Pala [1], José Avila [6,*] and Abdelkarim Ouerghi [1,*]

[1] Centre de Nanosciences et de Nanotechnologies, CNRS, Université Paris-Saclay, 91120 Palaiseau, France; cyrine.ernandes@universite-paris-saclay.fr (C.E.); lama.khalil@universite-paris-saclay.fr (L.K.); hugo.henck@gmail.com (H.H.); julien.chaste@c2n.upsaclay.fr (J.C.); fabrice.oehler@c2n.upsaclay.fr (F.O.); debora.pierucci@c2n.upsaclay.fr (D.P.); marco.pala@c2n.upsaclay.fr (M.P.)

[2] Department of Chemical and Materials Engineering, New Jersey Institute of Technology, 138 Warren Street, Newark, NJ 07103, USA; mz24@njit.edu

[3] Department of Physics and Astronomy, University of Pennsylvania, 209S 33rd Street, Philadelphia, PA 19104, USA; cjohnson@physics.upenn.edu

[4] Materials Science Institute of Madrid (ICMM), Spanish Scientific Research Council (CSIC), Cantoblanco, 28049 Madrid, Spain; mc.asensio@csic.es

[5] MATINEE: CSIC Research Associated Unit between the Institute of Materials Science of the Valencia University (ICMUV) and the ICMM, Cantoblanco, 28049 Madrid, Spain

[6] Synchrotron-SOLEIL, Université Paris-Saclay, Saint-Aubin, BP48, 91192 Gif sur Yvette, France

* Correspondence: jose.avila@synchrotron-soleil.fr (J.A.); abdelkarim.ouerghi@c2n.upsaclay.fr (A.O.)

Growth of $WS_2/SiO_2/Si(001)$: WS_2/SiO_2 samples were grown by chemical vapor deposition (CVD) in a 1” quartz tube furnace. The growth substrate (SiO_2/Si(001)) was placed in the center of the furnace and heated to 800 °C. A 25 mg sulfur pellet was placed on a piece of silicon and positioned upstream in the furnace such that its temperature was approximately 150 °C. Carrier gas (500 sccm N_2 and 15 sccm H_2) was used to bring sulfur vapor into the furnace for a 30 min growth period. A PMMA assisted method was employed to transfer the CVD grown WS_2 on epitaxial monolayer graphene on SiC. Typical single-crystal domains with an equilateral triangle shape were obtained by the CVD growth procedure. The WS_2 domains transferred onto the graphene retain their triangular shapes with lateral sizes of ~20 to ~200mm. To further clean the surface and interface of the WS_2/graphene, we annealed the samples at 350°C for 120 min in UHV (base pressure below P ~ 10^{-10}mbar).

Band structure of WS_2/graphene heterostructure: The nanoARPES measurements (spot seize about 600 nm) were conducted at the Antares beamline of Synchrotron SOLEIL (Saint-Aubin, France). We used linearly polarized photons of 100 eV and a hemispherical electron analyzer with vertical slits to allow band mapping. All ARPES experiments were done at low temperature (70 K).

DFT calculations: Band structures calculations were realized with the QUANTUM EPSRESSO code [35]. We adopted fully relativistic pseudopotentials and performed non-collinear simulations to include the spin-orbit interaction. For the exchange-correlation term we considered both the PBE and the HSE hybrid functional [36] to better estimate the band-gap energy. The self-consistent solution was obtained by adopting a 10x15x1 Monkhorst-Pack grid and a cutoff energy of 50 Ry. A vacuum space of 20 Å along the vertical direction was used to minimize the interaction between two adjacent sheets. Cell parameters and atomic positions were relaxed according to a convergence threshold for forces and energy of 10-3 and 10-4 (a.u,), respectively.

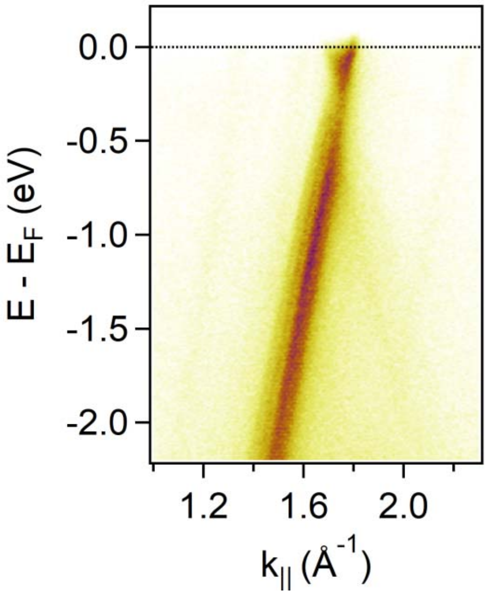

Figure S1. Typical Band structure of graphene layer on SiC(0001) (inside and outside of the WS_2).

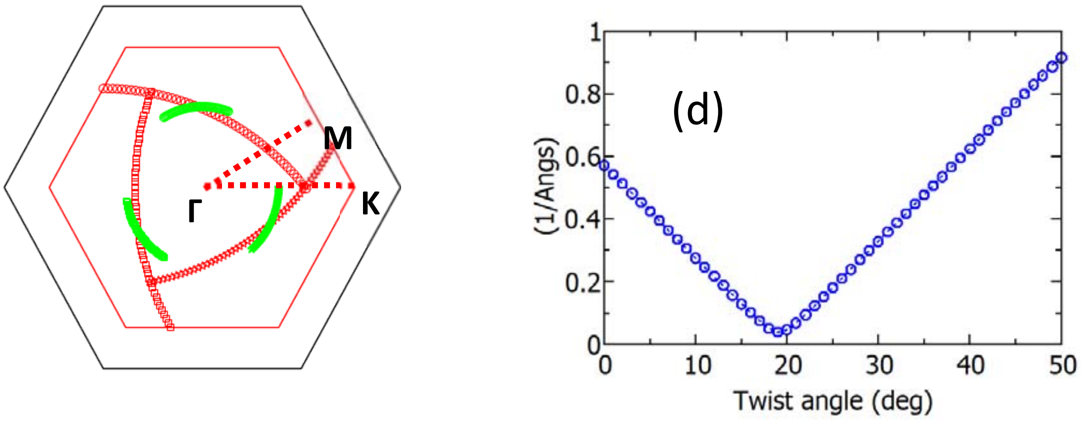

(a) **(b)**

Figure S2. (**a**) Band-structure calculations of the monolayer WS_2 along the high symmetry points of the Brillouin zone for different stress levels. (**b**) The evolution of the three wavevectors k_1^θ , k_2^θ and k_3^θ as a function of the twist angle θ. The hexagons represent the first Brillouin zone of (black) graphene, (red) WS_2 with θ = 0 and (magenta) WS_2 with θ = 20°. The green symbols represent the evolution of the Q points. (c) Distance between k_1^θ and Q as a function of θ.

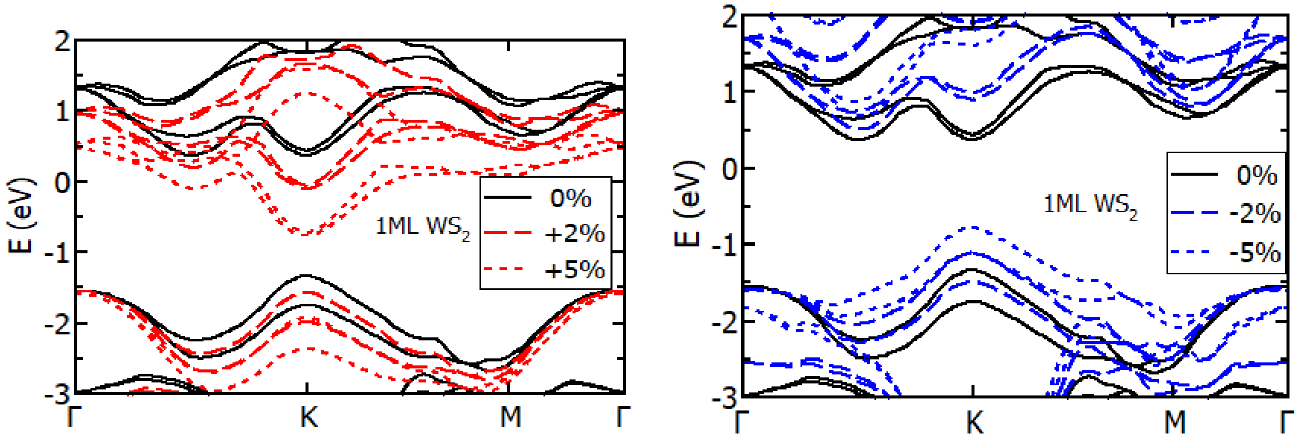

Figure S3. Evolution of the band-structure calculations of the monolayer WS_2 under strain.

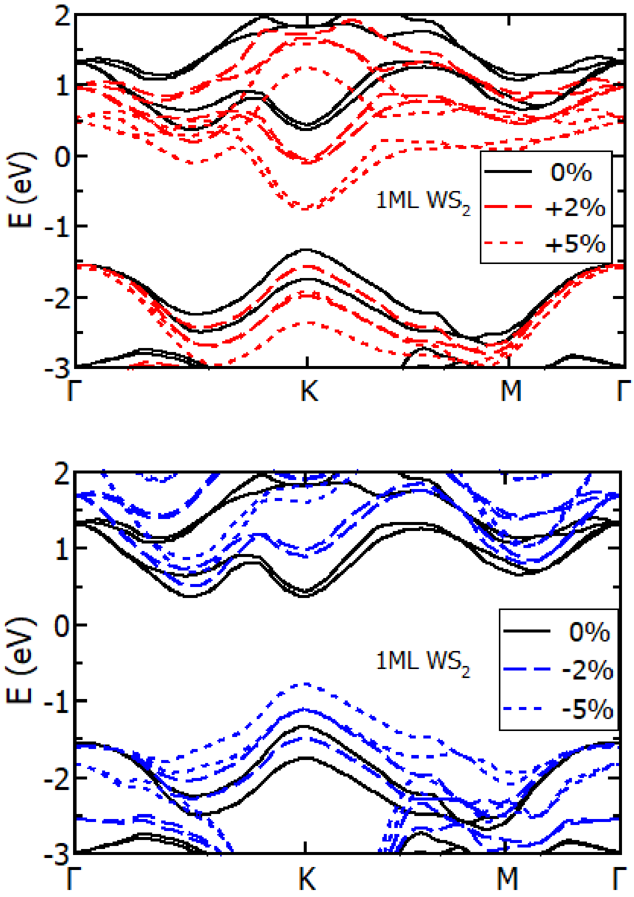

Figure S4. Evolution of the band-structure calculations of the monolayer WS_2 under strain.
